# Homophily, heterophily and the diversity of messages among decision-making individuals

**DOI:** 10.1098/rsos.180027

**Published:** 2018-04-11

**Authors:** Pouria Ramazi, James Riehl, Ming Cao

**Affiliations:** ENTEG, Faculty of Science and Engineering, University of Groningen, Nijenborgh 4, 9747 AG Groningen, The Netherlands

**Keywords:** evolutionary game theory, cooperation, homophily, heterophily

## Abstract

To better understand the intriguing mechanisms behind cooperation among decision-making individuals, we study the simple yet appealing use of preplay communication or cheap talk in evolutionary games, when players are able to choose strategies based on whether an opponent sends the same message as they do. So when playing games, in addition to pure cooperation and defection, players have two new strategies in this setting: homophilic (respectively, heterophilic) cooperation which is to cooperate (respectively, defect) only with those who send the same message as they do. We reveal the intrinsic qualities of individuals playing the two strategies and show that under the replicator dynamics, homophilic cooperators engage in a battle of messages and will become dominated by whichever message is the most prevalent at the start, while populations of heterophilic cooperators exhibit a more harmonious behaviour, converging to a state of maximal diversity. Then we take Prisoner’s Dilemma (PD) as the base of the cheap-talk game and show that the hostility of heterophilics to individuals with similar messages leaves no possibility for pure cooperators to survive in a population of the two, whereas the one-message dominance of homophilics allows for pure cooperators with the same tag as the dominant homophilics to coexist in the population, demonstrating that homophilics are more cooperative than heterophilics. Finally, we generalize an existing convergence result on population shares associated with weakly dominated strategies to a broadly applicable theorem and complete previous research on PD games with preplay communication by proving that the frequencies of all types of cooperators, i.e. pure, homophilic and heterophilic, converge to zero in the face of defectors. This implies homophily and heterophily cannot facilitate the long-term survival of cooperation in this setting, which urges studying cheap-talk games under other reproduction dynamics.

## 1. Introduction

*Homophily*, the tendency to interact with similar others, and *heterophily*, the tendency to interact with different others, are both observed in nature. Examples include flocking of similar birds [1], formation of clusters of companionships in zebras [2] and dolphins [3] based on sex and age similarity, and heterophilic formation of task-related ties in collaboration networks [4]. It is of great interest to understand the evolution of these two tendencies under different circumstances [5]. Evolutionary game theory, a powerful tool in understanding the evolution of cooperation in natural systems [6,7–13], provides a promising model for the study of homophily and heterophily, namely *cheap-talk games* [14]. Originally motivated by explaining how cooperation can be maintained in evolutionary Prisoner’s Dilemma (PD) [15], game theorists have proposed different modified game settings, a promising one of which is the introduction of cheap-talk games that allows a costless, non-binding, non-verifiable communication between the players before the game, a *preplay communication*. Players simultaneously send costless signals or messages to their opponents from a set available to each player before they play and consequently act based on the received messages [16]. A simple set-up is when each player treats similarly all received messages that are different from what the player sends. Homophily (respectively, heterophily) then is to cooperate (defect) only with those sending the same message as the player does, thus being also called *homophilic cooperation* (respectively, *heterophilic cooperation*) [17]. In addition to these two conditional decision rules in this set-up, there are two unconditional ones: *pure cooperation*, which is to always cooperate and *pure defection*, which is to always defect. Different nomenclatures have been used in the literature for the four types of players (table 1). Equivalent to games with preplay communication, in biology, one can think of individuals having recognizable phenotypes such as tags, on which they base their decisions [19,21]. Indeed, the *green beard* hypothesis postulates a gene for a simple recognition system. The gene simultaneously encodes (i) a conspicuous, heritable tag, such as a green beard, (ii) the ability to recognize the tag, and (iii) the tendency to cooperator against others with the same tag [20,22]. Although such genes have been reported [23–25], the green beard (tag-based cooperation) mechanism was often considered implausible as it requires a single gene for both altruism and a recognizable tag. Some more recent studies, however, place tag-based cooperation on a stronger theoretical ground where loosely coupled separate genes are considered and multiple tags can co-occur in the population [20,26].

**Table 1.** Different nomenclatures of the four types of players. The left column is used in this paper; the other columns are more evident in the literature and mainly used in tag-based frameworks [18–20].

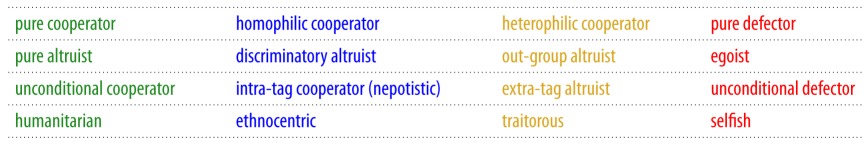

It is worth mentioning that a cheap-talk game differs from *signalling games* [27] where there is exactly one sender who does not act and only sends a signal, and exactly one receiver who does not send a signal and only acts according to the received message, resulting in just two types of players. Despite the difference, the literature on signalling games pose seemingly similar hypotheses to those for cheap-talk games, the most well known of which is that signals must be costly for cooperation to survive [28,29]. Another related yet different mechanism is *pre-commitment* where in addition to pure cooperation and defection, a player can propose her opponent to commit to cooperation before playing the game and is willing to pay a set-up cost; the opponent will be penalized for defection. Essentially modifying the pay-off matrix of the game, the mechanism is reported capable of promoting cooperation in PD games, even in the absence of repeated interactions, reputation effects and networks reciprocity in both pairwise [30] and group interactions [31]. In line with these works is the literature on *apology modelling* where only sufficiently sincere (costly) apologetic messages promote high levels of cooperation [32,33].

Several studies have investigated the asymptotic behaviour of individuals’ population shares associated with the above four decision rules under birth–death population dynamics [4,34]. Many others have studied the four types in a structured population and when PD is taken as the base game [18,35–39]. Particularly in [36], homophilic cooperators are shown to take over 0.75 of a lattice-structured population. It is argued in [40] that the dominance of homophilics in [36] is mainly due to the population structure not the tag mechanism (preplay communication). The corresponding simulation on a well-mixed population with random interactions and four tags shows that homophilics only take 0.23 of the population, whereas defectors take 0.61. However, still a considerable portion of the population is taken by non-pure defectors, leaving open the question of whether their survival, particularly that of homophilics, is due to random interactions and mutation. The simulation study [18] supports the so-called *direct* hypothesis for explaining the dominance of homophilics in structured populations: predominantly homophilics directly exploit predominantly pure cooperators along frontiers between homophilics and pure cooperators. This is in contrast with the little-supported *free-rider-suppression* hypothesis: predominantly homophilics are uniquely effective at suppressing predominantly free-riders (who defect against cooperation). In general, it seems that preplay communication does not help to maintain cooperation in well-mixed populations, unless the population exclusively comprises one (or two) of the type(s), e.g. only homophilics [41] (also see [42] and the negating argument [43]). In [19], for example, it is stated that altruism is lost when the tag and decision rule traits are always inherited together, which is the case with usual evolutionary game theoretical settings such as the replicator dynamics [44–46]. The claim, however, remains without a mathematical proof and is explained only via examples and simulations. Existing mathematical analyses on the asymptotic behaviour of population dynamics with preplay communication [47–50] do not completely solve this problem either. One only knows that if a strategy is strictly dominated in the base game of a cheap-talk game, e.g. cooperation in the PD game, its relative frequency goes to zero along any interior solution path to the replicator dynamics [16]. This implies that in the face of pure defectors, the population share of pure and homophilic cooperators goes to zero. So heterophilic cooperators are not ruled out, pleading for a more comprehensive analysis.

In this paper, we begin by uncovering the innate characteristic of well-mixed homophilic and heterophilic populations under the replicator dynamics in a general game setting. In particular, we find that populations of homophilic cooperators engage in a battle of messages (tags), and will become dominated by whichever message was most prevalent at the start, while populations of heterophilic cooperators exhibit a more harmonious behaviour, converging to a state of maximal message diversity. These results hold for the broad class of evolutionary games in which there is an advantage to mutual cooperation over defection. When engaged in a PD game, a mixture of both types of cooperators can result in a limit cycle, with each tag dominating for some fraction of time. Moreover, pure cooperators may coexist with homophilic cooperators but vanish in the presence of heterophilics. Finally, by developing a convergence theorem on weakly dominated strategies, we establish once and for all that defectors completely take over the population, leaving no room for cooperation of any type including pure, homophilic and heterophilic cooperators.

We consider a large population of individuals playing symmetric two-player games with two strategies: to ‘cooperate’, denoted by C and to ‘defect’, denoted by D, and with the pay-offs integrated in the pay-off matrix

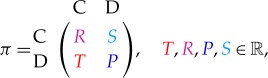
where 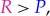, i.e. mutual cooperation exceeds mutual defection. We refer to this game as the *base game* *G* and are interested in the case when individuals have the ability to engage in some preplay communication, resulting in a *cheap-talk game* $G_{M}$. Namely, before playing the base game *G*, each player sends one costless message to her opponent, and simultaneously receives the message just sent by her opponent. The two preplay messages initiated by the two players are sent simultaneously and chosen from a finite set M={1,…,m},m≥2, of messages available to both players. The players may then base their strategies on the messages they have received, in the form of decision rules. We consider the case when messages different from that of the player herself are treated similarly, yielding the following four decision rules:

1. *C*, pure cooperation, under which the player always cooperates regardless of her opponent’s message;
2. C*, homophilic cooperation or homophily, under which the player cooperates if and only if her opponent’s message matches that of her own;
3. D*, heterophilic cooperation or heterophily, under which the player cooperates if and only if her opponent’s message is different from that of her own;
4. *D*, pure defection, under which the player always defects regardless of her opponent’s message.

Let $K=\{C,C^{*},D^{*},D\}$ be the set of decision rules. Then each individual sends a message i∈M and follows a decision rule X∈K, resulting in a cheap-talk pure strategy *X*_*i*_ that we characterize by the unit vector whose elements are all zero except for the (4(*i*−1)+*p*_*X*_)th element:

$$X_{i}={\left[\underset{4(i-1)+p_{X}}{\underbrace{0\;\ldots \;0\;1}}0\;\ldots 0\right]}^{T},$$

where $p_{X},X\in K,$ is defined as *p*_*C*_=1, *p*_C*_=2, *p*_D*_=3 and *p*_*D*_=4. Based on their strategies in the cheap-talk game, players earn pay-offs in the base game, which is captured by the *cheap-talk pay-off matrix* $\pi_{M}$ defined by

$$\pi_{M}={\left(\begin{array}{cccc} \pi ⊗\text{1} & \text{1}⊗\pi & \ldots & \text{1}⊗\pi \\ \text{1}⊗\pi & \pi ⊗\text{1} & \ldots & \text{1}⊗\pi \\ \vdots & \vdots & \ddots & \vdots \\ \text{1}⊗\pi & \text{1}⊗\pi & \ldots & \pi ⊗\text{1} \end{array}\right)}_{4m\times 4m},$$

where ⊗ denotes the Kronecker product and **1** the 2×2 all-one matrix. Then the pay-off an individual playing strategy *X*_*i*_, X∈K,i∈M, earns against another individual playing strategy *Y*_*j*_, Y∈K,j∈M, equals *u*(*X*_*i*_,*Y*_*j*_) where *u*, the *utility function*, is defined by $u(x,y)=x^{T}\pi_{M}y$ for $x,y\in R^{4m}$.

We study the evolution of individuals’ population shares under the replicator dynamics. Denote the population share of individuals playing strategy *X*_*i*_ by *x*_*X*_*i*__. Then the population state vector equals

$$x=\sum_{X\in K,i\in M}x_{X_{i}}X_{i}$$

and the average pay-off an *x*_*X*_*i*__-playing individual earns against the population equals *u*(*X*_*i*_,*x*). The evolution over time of the population share of individuals playing *X*_*i*_ is given by the replicator dynamics

1.1 $${\dot{x}}_{X_{i}}=[u(X_{i},x)-u(x,x)]x_{X_{i}},\;i=1,\ldots ,n.$$

Roughly speaking, comparing to the average population pay-off, the more an individual gains when playing against her opponents, the more she produces new offspring. The main goal of this paper is to study the asymptotic behaviour of the four types of individuals in different population mixtures under the dynamics (1.1). First, we consider a population of homophilic cooperators and then that of heterophilic cooperators.

**Theorem 1.1**

*Consider an exclusive population of homophilic cooperators under the dynamics (1.1). Then for any* i,j∈M*,
*

$$x_{C_{i}^{*}}(0)>x_{C_{j}^{*}}(0)\Rightarrow \lim_{t\to \infty }x_{C_{j}^{*}}(t)=0.$$

**Figure 1.**
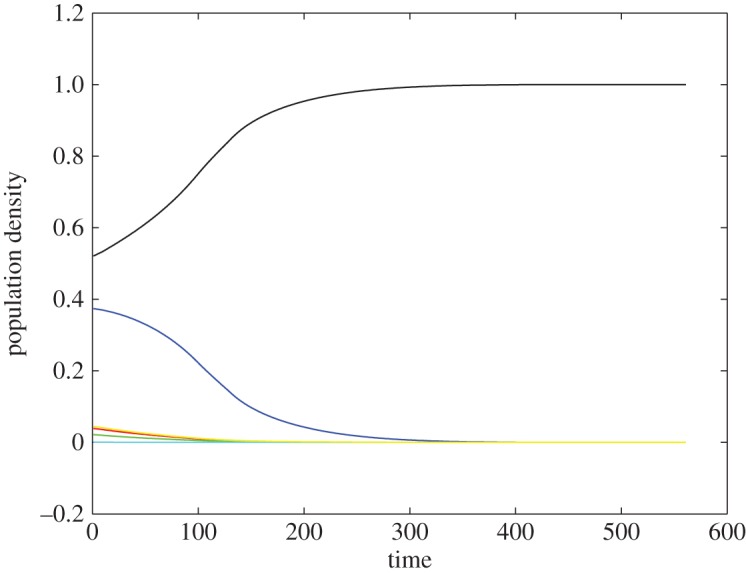
Evolution of homophilic cooperators. Different colours represent different messages. Those homophilic cooperators with the initially dominant message eventually take over the population.

All proofs of the theorems can be found in the *supplementary information*. As indicated by theorem 1.1, homophilic’s hostility towards outsiders results in a *battle of messages (tags)*, where a single message ends up taking over the entire population (figure 1). Not surprisingly, the winning message turns out to be the one that started with the greatest population share. The intuition behind this is that individuals sending the most populous message are also most likely to interact with cooperative opponents. The final population state is also robust to message mutations, meaning that any newly formed messages are quickly eliminated. In the rare case of having two or more most populous messages in the start, they will share the final population equally.

**Theorem 1.2**

*Consider an exclusive population of heterophilic cooperators under the dynamics (1.1). Then for any* i,j∈M*,
*

$$x_{D_{i}^{*}}(0),x_{D_{j}^{*}}(0)\neq 0\;\Rightarrow \;\lim_{t\to \infty }x_{D_{i}^{*}}(t)=\lim_{t\to \infty }x_{D_{j}^{*}}(t).$$

**Figure 2.**
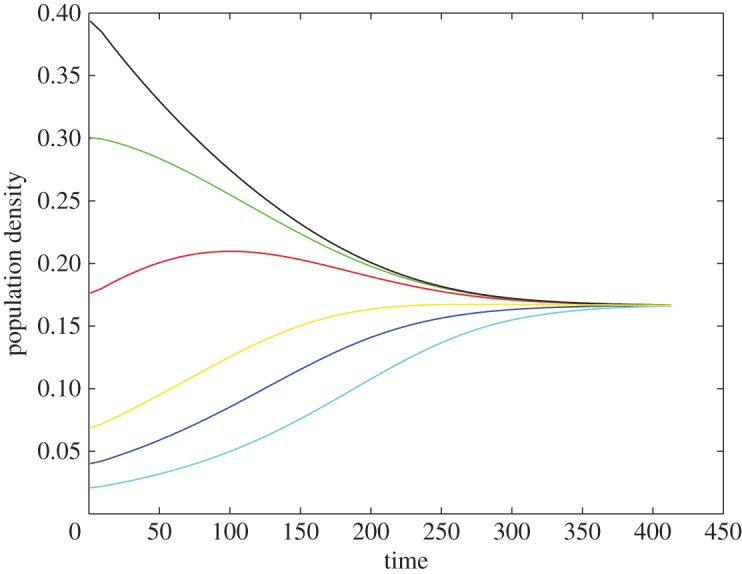
Evolution of heterophilic cooperators. Different colours represent different messages. Heterophilic cooperators with different messages equally share the population in the long run.

From theorem 1.2, in populations consisting exclusively of heterophilic cooperators, we observe the opposite phenomenon as that in the homophilic case, resulting in a *harmony of messages (tags)* (figure 2). Although diversity is a known property of heterophilic populations, it has not yet been shown that heterophily indeed drives the population to a *maximally balanced* state with all messages holding equal shares of the population. This occurs because members sending the least common message are now most likely to meet a cooperative opponent, while the converse is true for members sending the most common message. Hence there is a balancing effect putting upward pressure on those tags having below-average representation and downward pressure on those having above-average representation until the difference in population share between groups converges to zero. An interesting side effect occurs if a brand new message appears via mutation. This new message will be welcomed into the population but will grow only until it reaches the same population share as all the other tags. In this sense, heterophilic cooperators produce a population that is more tolerant than homophilic cooperators and will hence result in greater diversity. These results hold for any symmetric game in which the pay-offs for mutual cooperation are greater than those for mutual defection. This battle and harmony of messages has been reported in the simulation work [34], but in a death–birth process and different game set-up.

**Figure 3.**
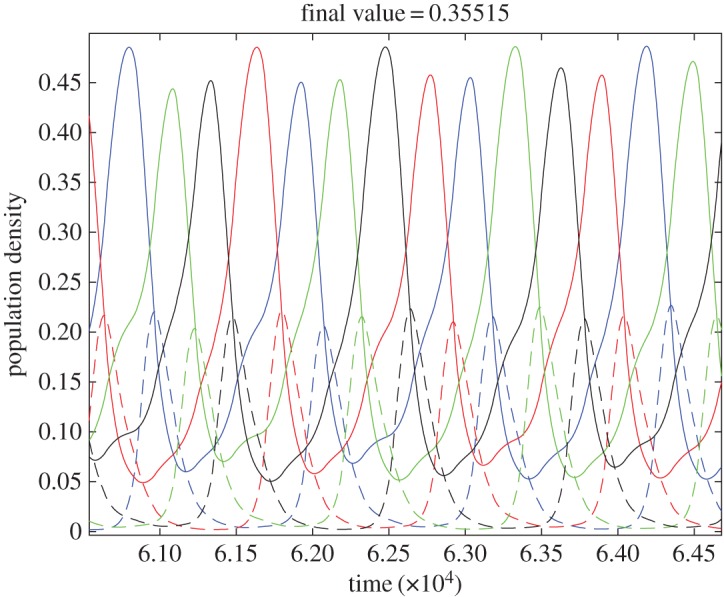
Co-evolution of homophilic and heterophilic cooperators. Different colours represent different messages. Solid and dashed lines denote homophilic and heterophilic cooperators. The two undergo non-ending cycles as time evolves.

To investigate how cooperative homophilics and heterophilics are, particularly in the face of pure cooperators and defectors, we choose the base game to be the challenging PD game, in which the pay-offs satisfy
1.2
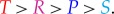
So far, we have seen that a population of homophilics will become dominated by a single tag, while a population of heterophilics converges to a maximally tag-balanced state. When these two types are mixed, one would expect a conflict to arise out of the simultaneous battle and harmony of messages, and indeed we observe just that—the population share of each tag exhibits large oscillations, which, depending on the pay-offs and the number of available tags, may persist indefinitely suggesting a heteroclinic cycle (figure 3), or instability may occur. For example, consider a homophilic cooperator with some rare message *i* appearing as a mutant in a population. This mutant will defect against all messages not equal to *i*, which hold the vast majority of the population. Then, if the average individual pay-off in the population is below some threshold, the mutant will invade and eventually wipe out all existing messages, resulting in a uniform population of homophilics sending message *i*. Now a heterophilic cooperator sending the same message *i* can invade this population as it defects against its own tag, but the homophilics that make up the population cooperate in return with this new mutant. As a result, the population will eventually be completely taken over by heterophilics with message *i*, which again creates an opening for a homophilic cooperator with a different rare tag *j* to invade, and the process repeats indefinitely among different messages, resulting in a heteroclinic cycle.

Now if we consider a population that contains both homophilic and pure cooperators, an interesting phenomenon occurs. Having various messages but no preference in others, the pure cooperators are subject to the prejudices of the other homophilic cooperators and thus the battle of messages remains in effect. Once again, the message(s) occupying the largest portion of the initial population will prevail as indicated in the following theorem, and the final population will generally contain both homophilic and pure cooperators sending this message. Let $x_{X},X\in K,$ and $x_{i},i\in M,$ denote the population share of individuals following decision rule *X* and sending message *i*, respectively. Clearly $x_{X}=\sum_{j\in M}x_{X_{j}},x_{i}=\sum_{Y\in K}x_{Y_{i}}$.

**Theorem 1.3**

*Consider an exclusive population of pure and homophilic cooperators where for every message* i∈M*x*_C*_i__>0. *Then under the dynamics (1.1) and when (1.2) is fulfilled, at least one of the following holds*

$$\lim_{t\to \infty }x_{C}(t)=0$$

*or
*

$$\exists i\in M:\lim_{t\to \infty }x_{i}(t)=1.$$

A similar result has been claimed under the discrete-time replicator dynamics with only two available messages in [51]. In fact, homophilic cooperators are the only types with which pure cooperators can coexist in the PD. Heterophilic cooperators still defect against pure cooperators sending the same message, so in a mixed population of these two types engaged in a PD, the heterophilic cooperators will wipe out the pure cooperators as they converge to the balanced state:

**Theorem 1.4**

*Consider an exclusive population of pure and heterophilic cooperators where for every message* i∈M*, if x*_*C*_*i*__>0, *then x*_D*_i__>0. *Then under the dynamics (1.1) and when (1.2) is fulfilled,*

$$\lim_{t\to \infty }x_{C}(t)=0.$$

It is worth mentioning that pure cooperators also do not survive in a population of the three types of individuals (see electronic supplementary material, Proposition S1). Setting aside all interactions between homophilic, heterophilic and pure cooperators, defectors still play their dominance role in this game. The following theorem mathematically supports this claim, which is a negative answer to the posed hypothesis, that preplay communication may by itself facilitate the emergence of cooperation in the PD game, and hence highlights the emergence of other reproductive dynamics such as the chromodynamics [26] for altruism to evolve. Cooperation is facilitated also when structure is introduced to the population, viscosity is considered in the model [36], or indirect reciprocity is allowed, i.e. an individual with message *i* may detect the type of her opponents, and hence, defect against those who defect against players with message *i* (not because her opponents will defect against herself as they may hardly meet more than once, but as her opponents will defect against her fellows with the same tag *i*) [1]. The theorem also postulates that the survival of homophilics in Janson’s simulation [40] is not their intrinsic property, but perhaps caused by random interactions or mutation.

**Theorem 1.5**

*Consider a population where for each message, the population share of pure defector sending that message is non-zero. Then under the dynamics (1.1) and when (1.2) is fulfilled, the population share of all types but the pure defectors converges to zero, i.e.
*

$$\lim_{t\to \infty }x_{D^{*}}(t)=\lim_{t\to \infty }x_{C^{*}}(t)=\lim_{t\to \infty }x_{C}(t)=0.$$

What enables us to prove theorem 1.5, is the establishment of a fundamental convergence result that applies more broadly to any normal two-player game, motivating us to frame it in general terms. Consider a normal symmetric two-player game with *n* strategies and a pay-off matrix $A\in R^{n\times n}$. Define the simplex

$$\Delta :=\left\{x\in R^{n}\;|\;\sum_{i=1}^{n}x_{i}=1,0\leq x_{i}\leq 1\right\}.$$

Let *e*^*i*^,*i*=1…,*n*, be the *n*th column of the *n*×*n* identity matrix, and define $E=\{e^{i}\;|\;i=1,\ldots ,n\}.$ Each *e*^*i*^ is a pure strategy. A *mixed strategy* is a vector *x*∈Δ, and can be obtained by some convex combination of the vertices of Δ. Consider a non-empty subset H⊆E. The convex hull of H is a *face* of Δ and is denoted by Δ(H) [16]. We say that strategy *x* *weakly dominates strategy *y* in Δ(H)*, if (i) x,y∈Δ(H), (ii) *u*(*x*,*z*)≥*u*(*y*,*z*) for all z∈Δ(H) and (iii) *u*(*x*,*r*)>*u*(*y*,*r*) for at least one r∈Δ(H). This is a reformulation of weak dominance in the whole simplex. Besides being a strategy, a vector *x*∈Δ can also represent a population vector whose entries *x*_*i*_ denote the population share of individuals playing strategy *i*. Then the replicator dynamics can be written as

1.3 $${\dot{x}}_{i}=[u(e^{i},x)-u(x,x)]x_{i}\;i=1,\ldots ,n.$$

For ease of analysis, we propose the following notation. Given a pure strategy *v*=*e*^*i*^ for some *i*∈{1,…,*n*} define *x*_*v*_:=*x*_*i*_. For example, when *v*=[0 1 0], we have that *x*_*v*_=*x*_2_. The following result, which we have reformulated to make it applicable to Δ(H) instead of just Δ, is a classical result on convergence of weakly dominated strategies under the dynamics (1.3).

**Proposition 1.6 ([16] weakly dominated strategies).**

*Suppose that a pure strategy* *a* *is weakly dominated by some strategy* *y* in Δ(H). *If* *u*(*y*,*b*)>*u*(*a*,*b*) *for some pure strategy* *b*, *then the following holds under the dynamics* (*1.3*) *and for any initial state* x(0)∈int(Δ(H)):

$$\lim_{t\to \infty }x_{a}(t)=0\;\vee \;\lim_{t\to \infty }x_{b}(t)=0.$$

Together with other convergence results [52], the proposition can be used for analysing the asymptotic behaviour of the replicator dynamics. Now let P and H be two non-empty subsets of E such that H⊂P. It often happens that a pure strategy *a* is not weakly dominated in the face Δ(P), but it is weakly dominated in the boundary face Δ(H). Then of course lemma 1.6 can be applied whenever the initial population state *x*(0) belongs to int(Δ(H)), but what if *x*(0) belongs to int(Δ(P)) and not to int(Δ(H))? Intuitively, a similar result to that of proposition 1.6 should hold when we know that *x*_*j*_→0 for all pure strategies *j* that are in P but not in H. We confirm this in the following main result.

**Theorem 1.7**

*Let* P *be a set of pure strategies, and consider a non-empty subset* H *of it. Suppose that a pure strategy a is weakly dominated by some strategy y in the face* Δ(H)*. Also let u(y,b)>u(a,b) for a pure strategy* b∈H*. If
*

1.4 $$\lim_{t\to \infty }x_{j}=0\;\forall j\in P-H,$$

*then under the dynamics (1.1) and for any* x(0)∈int(Δ(P))*, it holds that
*

1.5 $$\lim_{t\to \infty }x_{a}=0\;\vee \;\lim_{t\to \infty }x_{b}=0.$$

The results in this paper reveal the intrinsic qualities of homophilic (indoor) and heterophilic (outdoor) cooperators. ‘Only my phenotype (message) should survive’ is the tendency homophilics show leading indeed to only one group surviving in their exclusive population. This suggests the presence of homophily in populations where one phenotype dominates the population share. It is also because of this attitude that homophilics are the only type of individuals with whom pure cooperators may survive, under the PD. On the other hand, heterophilics exhibit a ‘welcoming’ attitude leading to diversity and balance in their exclusive population. This suggests the presence of heterophily in highly phenotype-balanced populations. A population mixture of these two types results in oscillations that can either persist as in a heteroclinic cycle, or become unstable. This periodic behaviour may be helpful when seeking populations exhibiting both homophily and heterophily. The last result here is that if pure defectors are present, there is no room for cooperation of any kind in the PD.

## Supplementary Material

Supplementary Material

## Data accessibility

Proofs of the results are available as electronic supplementary material and simulation codes are available upon request to the corresponding author.
