# Supplementary material for: Homophily, heterophily and the diversity of messages among decision-making individuals
Source: R Soc Open Sci. 2018 Apr 11;5(4):180027. doi: 10.1098/rsos.180027 (PMC5936958; doi:10.1098/rsos.180027)
Supplement: Supplementary Material [file rsos180027supp1.pdf]

Supplementary Material for Homophily, Heterophily, and the Diversity of Messages Among Decision-Making Individuals

Pouria Ramazi[†], James Riehl[†], and Ming Cao[†]

[†]ENTEG, Faculty of Mathematics and Natural Sciences, University of Groningen, The Netherlands, p.ramazi@gmail.com, {j.r.riehl,m.cao}@rug.nl

This document contains the proofs for the theorems in the paper Homophily, Heterophily, and the Diversity of Messages Among Decision-Making Individuals.

We first observe that for any initial condition $x^0 \in \Delta_{\mathcal{M}}$, there exists a unique, continuous solution to the replicator dynamics (1) in the paper for all $t \geq 0$, which is continuously differentiable with respect to time [1, Proposition 3.20]. This basic property enables us to carry out further proofs as follows.

Proofs of Theorems 1 and 2

Theorem 1: Consider an exclusive population of homophilic cooperators under the dynamics (1). Then for any $i, j \in \mathcal{M}$,

$$x_{C_i^*}(0) > x_{C_j^*}(0) \Rightarrow \lim_{t\to\infty} x_{C_j^*}(t) = 0.$$

Proof: Should $x_{C_j^*} = 0$, the result is trivial, so assume otherwise. Then $0 < x^0_{C_j^*} < 1$, and in fact following some standard continuity argument, one can check that $0 < x_{C_j^*}(t) < 1$ for all $t \geq 0$. So we can define the ratio $\frac{x_{C_i^*}}{x_{C_j^*}}$ of the population shares and calculate its time derivative

$$\frac{d}{dt}\left(\frac{x_{C_i^*}}{x_{C_j^*}}\right) = [u(x_{C_i^*}, x) - u(x, x)]\frac{x_{C_i^*}}{x_{C_j^*}}$$

$$\Rightarrow \frac{d}{dt}\left(\frac{x_{C_i^*}}{x_{C_j^*}}\right) = (R - P)[x_{C_i^*} - x_{C_j^*}]\frac{x_{C_i^*}}{x_{C_j^*}}. \tag{S1}$$

Since $R > P$ and $x_{C_i^*}(0) > x_{C_j^*}(0)$, the right-hand side of (S1) is positive at $t = 0$, so $x_{C_i^*}/x_{C_j^*}$ increases at t_0, which in turn makes $x_{C_i^*} - x_{C_j^*}$ increase and so does the infinitesimal increment of $x_{C_i^*}/x_{C_j^*}$. Repeating this argument, we know that $x_{C_i^*}/x_{C_j^*}$ keeps increasing nontrivially for the entire evolution since $x_{C_i^*}$ and $x_{C_j^*}$ are well defined and $x_{C_j^*} > 0$ for all t. So

$$\lim_{t\to\infty} \frac{x_{C_i^*}(t)}{x_{C_j^*}(t)} = \infty.$$

On the other hand, since $0 < x_{C_i^*}, x_{C_j^*} < 1$, this implies $\lim_{t\to\infty} x_{C_j^*}(t) = 0$, which completes the proof. ∎

Theorem 2: Consider an exclusive population of heterophilic cooperators under the dynamics (1). Then for any $i, j \in \mathcal{M}$,

$$x_{D_i^*}(0), x_{D_j^*}(0) \neq 0 \Rightarrow \lim_{t\to\infty} x_{D_i^*}(t) = \lim_{t\to\infty} x_{D_j^*}(t).$$

Proof: Similar to (S1) we obtain

$$\frac{d}{dt}\left(\frac{x_{D_i^*}}{x_{D_j^*}}\right) = -(R - P)[x_{D_i^*} - x_{D_j^*}]\frac{x_{D_i^*}}{x_{D_j^*}}.$$

One can check that the solution $x_{D_i^*} = x_{D_j^*}$ is globally asymptotically stable in the zone $0 < x_{D_i^*} = x_{D_j^*} < 1$ under the above differential equation, which completes the proof. ∎

Remark 1: Indeed there is a compelling one-to-one correspondence between the dual trajectories of the replicator dynamics starting from $\Delta_{\mathcal{M}}(C^*)$ and $\Delta_{\mathcal{M}}(D^*)$ respectively: they can be taken as the same trajectory, one flows forwards and the other backwards in time.

Proofs of Theorems 3, 4 and 5 and statement of Proposition S1

We first note that Proposition 1, Theorem 6 and the notion of a face, all mentioned in the end of the main article, will be used here. Moreover, a typical cheap-talk payoff matrix can help to understand the dominance relationships and would be

$$\begin{array}{c} \\ C_1 \\ C_1^* \\ D_1^* \\ D_1 \\ \\ C_2 \\ C_2^* \\ D_2^* \\ D_2 \end{array}
\begin{array}{c}
\begin{array}{cccccccccc} C_1 & C_1^* & D_1^* & D_1 & & C_2 & C_2^* & D_2^* & D_2 \end{array} \\
\left(\begin{array}{cccccccccc}
R & R & S & S & | & R & S & R & S \\
R & R & S & S & | & T & P & T & P \\
T & T & P & P & | & R & S & R & S \\
T & T & P & P & | & T & P & T & P \\
- & - & - & - & - & - & - & - & - \\
R & S & R & S & | & R & R & S & S \\
T & P & T & P & | & R & R & S & S \\
R & S & R & S & | & T & T & P & P \\
T & P & T & P & | & T & T & P & P
\end{array}\right)
\end{array}. \tag{S2}$$

Theorem 3: Consider an exclusive population of pure and homophilic cooperators where for every message $i \in \mathcal{M}$, $x_{C_i^*} > 0$. Then under the dynamics (1) and when (2) is fulfilled, at least one of the followings holds

$$\lim_{t\to\infty} x_C(t) = 0$$

or

$$\exists i \in \mathcal{M} : \lim_{t\to\infty} x_i(t) = 1.$$

Proof: Based on the population in question, $x(0) \in \operatorname{int}(\Delta(\mathcal{S}))$ for some $\mathcal{S} \subseteq \{X_j \,|\, X \in \{C, C^*\}, j \in \mathcal{M}\}$ where $\mathcal{S} \ni C_i^*$ for all $i \in \mathcal{M}$. Consider the reduced cheap-talk payoff matrix corresponding to decision rules C and C^*. A typical case would be

$$\begin{array}{c} \\ C_1 \\ C_1^* \\ \\ C_2 \\ C_2^* \end{array} \begin{array}{c} \begin{array}{ccccc} C_1 & C_1^* & & C_2 & C_2^* \end{array} \\ \begin{pmatrix} R & R & | & R & S \\ R & R & | & T & P \\ - & - & - & - & - \\ R & S & | & R & R \\ T & P & | & R & R \end{pmatrix} \end{array}.$$

For any $i \in \mathcal{M}$, C_i is weakly dominated by C_i^* in $\Delta(\mathcal{S})$. In addition, for any $j \in \mathcal{M}, j \neq i$,

$$u(C_i^*, C_j^*) > u(C_i, C_j^*) \qquad \text{and} \qquad u(C_i^*, C_j) > u(C_i, C_j).$$

Hence, in view of Proposition 1, at least one of the followings holds:

$$\lim_{t\to\infty} x_{C_i}(t) = 0$$

or

$$\lim_{t\to\infty} x_{C_j}(t) = \lim_{t\to\infty} x_{C_j^*}(t) = 0.$$

Repeating this argument for all other $j \in \mathcal{M}, j \neq i$, we acquire

$$\lim_{t\to\infty} x_{C_i}(t) = 0 \tag{S3}$$

or

$$\lim_{t\to\infty} x_{C_j}(t) = \lim_{t\to\infty} x_{C_j^*}(t) = 0 \quad \forall j \in \mathcal{M} - \{i\} \;\Rightarrow\; \lim_{t\to\infty} x_i(t) = 1. \tag{S4}$$

Since at least one of (S3) and (S4) takes place for every $i \in \mathcal{M}$, we conclude that at least one of the following two will happen

$$\forall i \in \mathcal{M} \quad \lim_{t\to\infty} x_{C_i}(t) = 0$$

or

$$\exists i \in \mathcal{M} : \lim_{t\to\infty} x_i(t) = 1,$$

which leads to the proof. ■

Theorem 4: Consider an exclusive population of pure and heterophilic cooperators where for every message $i \in \mathcal{M}$, if $x_{C_i} > 0$, then $x_{D_i^*} > 0$. Then under the dynamics (1) and when (2) is fulfilled,

$$\lim_{t\to\infty} x_C(t) = 0.$$

Proof: Based on the population in question, $x(0) \in \operatorname{int}(\Delta(\mathcal{S}))$ for some $\mathcal{S} \subseteq \{X_j \,|\, X \in \{C, D^*\}, j \in \mathcal{M}\}$ where $\mathcal{S} \ni D_i^*$ if $\mathcal{S} \ni C_i$, for all $i \in \mathcal{M}$. Consider the reduced cheap-talk payoff matrix corresponding to decision rules C and D^*. A typical case would be

$$\begin{array}{c} \\ C_1 \\ D_1^* \\ \\ C_2 \\ D_2^* \end{array} \begin{array}{c} \begin{array}{ccccc} C_1 & D_1^* & & C_2 & D_2^* \end{array} \\ \begin{pmatrix} R & S & | & R & R \\ T & P & | & R & R \\ - & - & - & - & - \\ R & R & | & R & S \\ R & R & | & T & P \end{pmatrix} \end{array}.$$

For any $i \in \mathcal{M}$, C_i is weakly dominated by D_i^* in $\Delta(\mathcal{S})$. In addition, for any $j \in \mathcal{M}, j \neq i$,

$$u(D_i^*, C_j) > u(C_i, C_j).$$

Hence, in view of Proposition 1,

$$\lim_{t\to\infty} x_{C_j}(t) = 0$$

The proof then follows the fact that the above equation holds for every $i \in \mathcal{M}$. ■

Proposition S1: Consider an exclusive population of pure, homophilic and heterophilic cooperators where for every message $i \in \mathcal{M}$, if $x_{C_i} > 0$, then $x_{D_i^*} > 0$. Then under the dynamics (1) and when (2) is fulfilled,

$$\lim_{t\to\infty} x_C(t) = 0.$$

Proof: Based on the population in question, $x(0) \in \mathrm{int}(\Delta(\mathcal{S}))$ for some $\mathcal{S} \subseteq \{X_j \,|\, X \in \{C, D^*\}, j \in \mathcal{M}\}$ where $\mathcal{S} \ni D_i^*$ if $\mathcal{S} \ni C_i$, for all $i \in \mathcal{M}$. Consider the reduced cheap-talk payoff matrix corresponding to decision rules C and D^*. A typical case would be

$$\begin{array}{c} \\ C_1 \\ C_1^* \\ D_1^* \\ \\ C_2 \\ C_2^* \\ D_2^* \end{array} \begin{array}{c} \begin{array}{ccccccc} C_1 & C_1^* & D_1^* & & C_2 & C_2^* & D_2^* \end{array} \\ \left(\begin{array}{ccccccc} R & R & S & | & R & S & R \\ R & R & S & | & T & P & T \\ T & T & P & | & R & S & R \\ - & - & - & - & - & - & - \\ R & S & R & | & R & R & S \\ T & P & T & | & R & R & S \\ R & S & R & | & T & T & P \end{array}\right) \end{array}.$$

The rest of the proof is the same as that of Theorem 4. ■

Theorem 5: Consider a population where for each message, the population share of pure defector sending that message is nonzero. Then under the dynamics (1) and when (2) is fulfilled, the population share of all types but the pure defectors converges to zero, i.e.,

$$\lim_{t\to\infty} x_{D^*}(t) = \lim_{t\to\infty} x_{C^*}(t) = \lim_{t\to\infty} x_C(t) = 0.$$

Proof: Based on the population in question, $x(0) \in \mathrm{int}(\Delta(\mathcal{S}))$ for some $\mathcal{S} \subseteq \{X_j \,|\, X \in \mathcal{K}, j \in \mathcal{M}\}$ where $\mathcal{S} \ni D_i$ for all $i \in \mathcal{M}$. Consider the reduced cheap-talk payoff matrix corresponding to decision rules C and D^*. A typical case is presented at (S2). For any $i \in \mathcal{M}$, C_i is weakly dominated by D_i in $\Delta(\mathcal{S})$. In addition,

$$u(D_i, C_i) > u(C_i, C_i).$$

Hence, in view of Proposition 1,

$$\lim_{t\to\infty} x_{C_i}(t) = 0 \quad \forall i \in \mathcal{M} \tag{S5}$$

Similarly, for any $i \in \mathcal{M}$, C_i^* is weakly dominated by D_i in $\Delta(\mathcal{S})$. In addition,

$$u(D_i, C_i^*) > u(C_i^*, C_i^*).$$

Hence, in view of Proposition 1,

$$\lim_{t\to\infty} x_{C_i^*}(t) = 0 \quad \forall i \in \mathcal{M} \tag{S6}$$

Now define the set $\bar{\mathcal{S}} = \mathcal{S} - \{X_i \,|\, X \in \{C, C^*\}, i \in \mathcal{M}\}$. For any $i \in \mathcal{M}$, there exists a sufficiently small $\epsilon > 0$ such that the mixed strategy $y = (1-\epsilon)D_i + \epsilon D_j$ for some $j \neq i$ weakly dominates D_i^* in $\Delta(\bar{\mathcal{S}})$. In addition,

$$u(y, D_i^*) > u(D_i^*, D_i^*) \qquad \forall i \in \mathcal{M}.$$

Hence, by taking $\mathcal{P} = \mathcal{S}$ and $\mathcal{H} = \bar{\mathcal{S}}$, from Theorem 6 we obtain

$$\lim_{t\to\infty} x_{C_i^*}(t) = 0 \quad \forall i \in \mathcal{M}. \tag{S7}$$

By summarizing (S5) to (S7), we arrive at the proof. ■

Proof of Theorem 6

Theorem 6: Let $\mathcal{P}$ be a set of pure strategies, and consider a nonempty subset $\mathcal{H}$ of it. Suppose that a pure strategy a is weakly dominated by some strategy y in the face $\Delta(\mathcal{H})$. Also let $u(y, b) > u(a, b)$ for a pure strategy $b \in \mathcal{H}$. If

$$\lim_{t\to\infty} x_j = 0 \qquad\qquad \forall j \in \mathcal{P} - \mathcal{H}, \tag{S8}$$

then under the dynamics (1) and for any $x(0) \in \mathrm{int}\,(\Delta(\mathcal{P}))$, it holds that

$$\lim_{t\to\infty} x_a = 0 \quad \vee \quad \lim_{t\to\infty} x_b = 0. \tag{S9}$$

Proof: Some ideas from the proof of Proposition 3.2 in [1] are used here. For each pure strategy j, let $\gamma_j = u(y - e^a, e^j)$. Since a is weakly dominated by y in $\Delta(\mathcal{H})$, it holds that $\gamma_j \geq 0, \forall j \in \mathcal{H}$. Moreover, $\gamma_b > 0$ because of $u(y, b) > u(a, b)$. Define the function $v_a : \mathrm{int}\,(\Delta(\mathcal{P})) \to \mathbb{R}$ by

$$v_a(x) := \log(x_a) - \sum_{j\in\mathcal{P}} y_j \log(x_j).$$

Clearly v_a is differentiable and its time derivative equals

$$\begin{aligned}\frac{d}{dt}v_a(x) &= \frac{\dot{x}_a}{x_a} - \sum_{j\in\mathcal{P}} \frac{y_j \dot{x}_j}{x_j} \\ &= u(e^a - x, x) - \sum_{j\in\mathcal{P}} y_j u(e^j - x, x) \\ &= u(e^a - x, x) - u(y - x, x) = -u(y - e^a, x) \\ &= -\sum_{j\in\mathcal{P}} u(y - e^a, e^j)x_j = -\sum_{j\in\mathcal{P}} \gamma_j x_j \\ &= -\sum_{j\in\mathcal{H}\cap\mathcal{P}} \gamma_j x_j - \sum_{j\in\mathcal{P}-\mathcal{H}} \gamma_j x_j \\ &\le -\gamma_b x_b - \sum_{j\in\mathcal{P}-\mathcal{H}} \gamma_j x_j.\end{aligned}$$

Hence,

$$v_a(x) \le v_a(x^0) - \gamma_b \int_0^t x_b(\tau)d\tau + \sum_{j\in\mathcal{P}-\mathcal{H}} \gamma_j \int_0^t x_j(\tau)d\tau.$$

Because of (S8) and the fact that all $x_j(t)$s are confined to $[0,1]$ for all $t \ge 0$, each integral $\int_0^t x_j(\tau)d\tau, j \in \mathcal{P} - \mathcal{H}$, converges to some constant α_j. On the other hand, since $0 \le x_b(t) \le 1$ for all $t \ge 0$, the integral $\int_0^t x_b(\tau)d\tau$ either converges to $+\infty$ or to some constant α_b. In the first case, since $\gamma_b > 0$ it holds that $v_a \to -\infty$ and hence $x_a \to 0$, according to the definition of v_a. In the second case, by the uniform continuity of x_b it can be shown that $x_b \to 0$, which completes the proof. ■

References

[1] J. W. Weibull, *Evolutionary Game Theory*. MIT Press, 1997.
